# Supplementary figures and images for: Differential immunomodulation of porcine bone marrow derived dendritic cells by E. coli Nissle 1917 and β-glucans
Source: PLoS One. 2020 Jun 19;15(6):e0233773. doi: 10.1371/journal.pone.0233773 (PMC7304589; doi:10.1371/journal.pone.0233773)

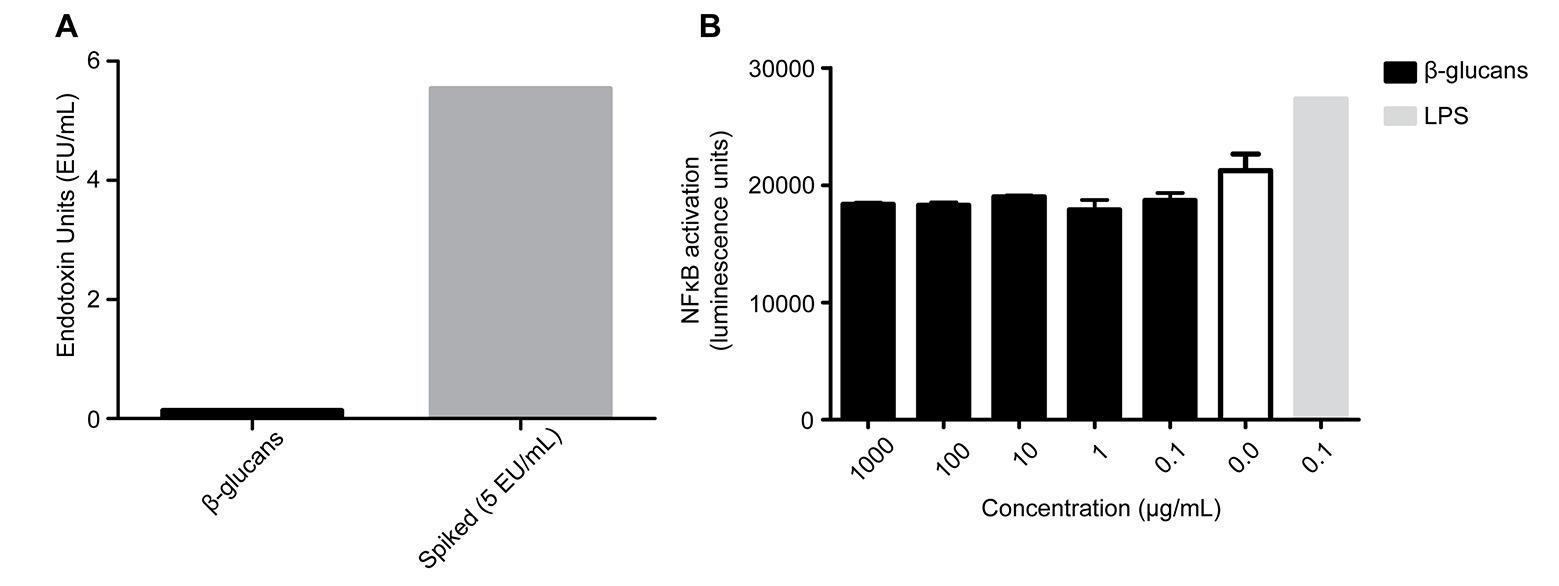

Supplement: S1 Fig — (A) 100 μg/mL β-glucans (MacroGard®) was tested for LPS contamination using a recombinant factor C LAL assay preparation. A 5 EU spiked control was included (n = 1). (B) Different concentrations (1 mg/mL– 0.1 μg/mL) of commercial β-glucans (MacroGard®) and LPS (100 pg/mL) were tested for their NF-κB activation via hTLR4 (n = 3). (TIF) [file pone.0233773.s001.tif]

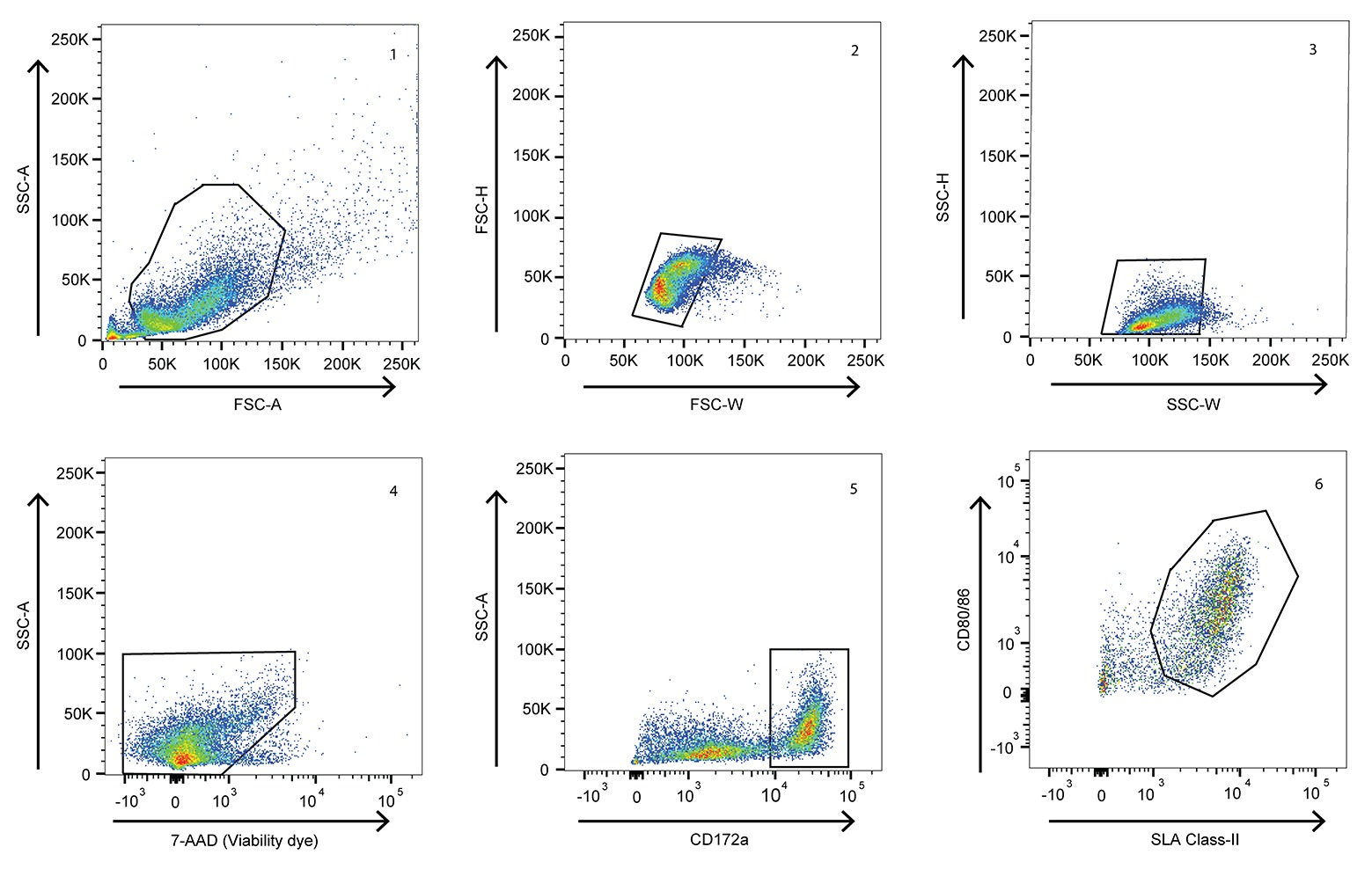

Supplement: S2 Fig — Gating strategy following multicolour flow cytometry staining using Abs against CD172a, SLA Class-II and CD80/86. Cells showing high forward scatter (FSC-A) and side scatter (SSC-A) profiles were gated, followed by the selection of single cells (FSC-W/H and SSC-W/H) and viable cells (SSC-A/7-AAD). Among these cells, BMDCs were defined as the CD172a+/high cells (SSC-A/CD172a) expressing SLA Class-II and CD80/86. (TIF) [file pone.0233773.s002.tif]

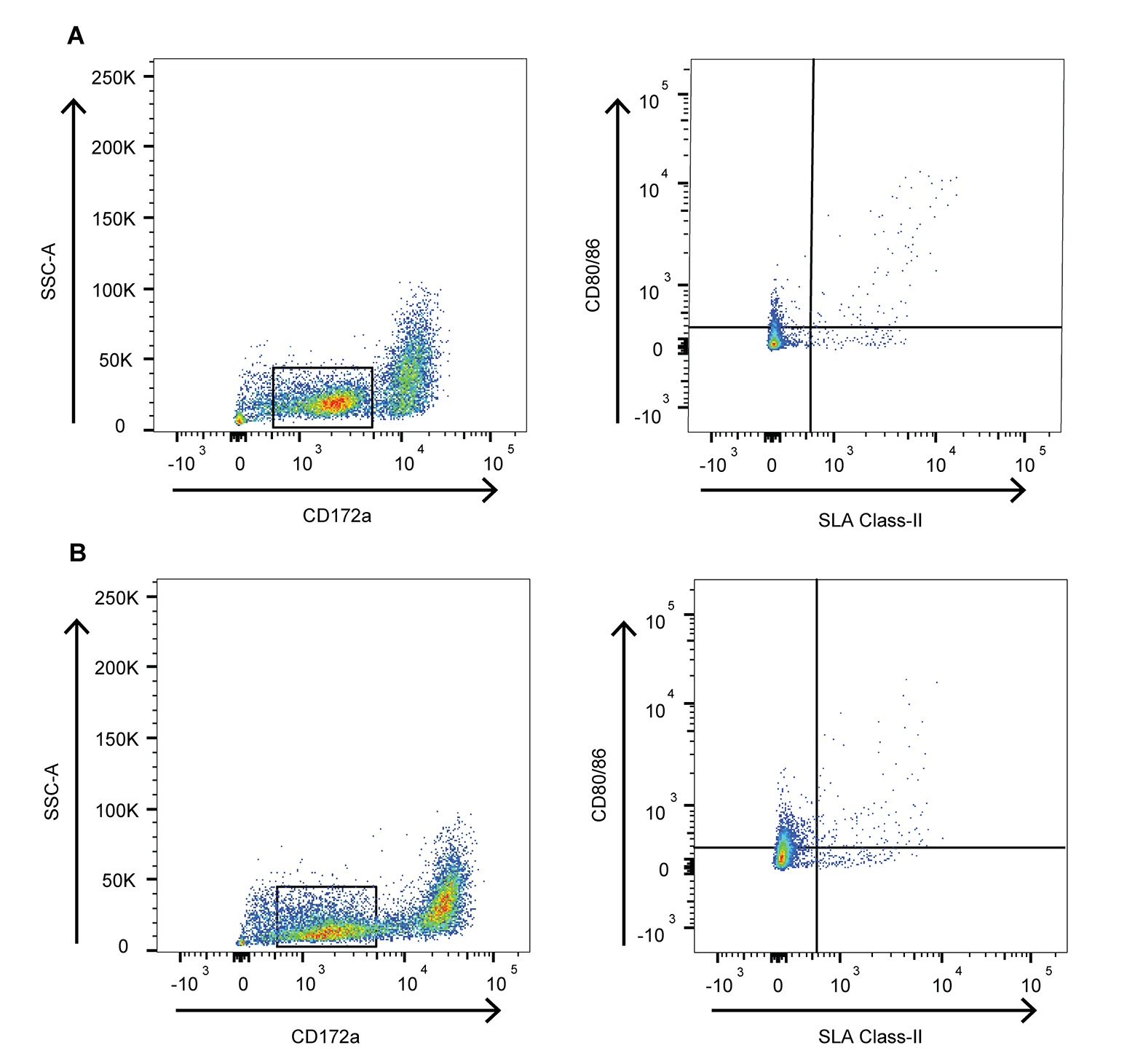

Supplement: S3 Fig — Gating strategy of (A) frhBMDCs and (B) cryoBMDCs following multicolour flow cytometry staining using Abs against CD172a, SLA Class-II and CD80/86. The CD172a+/- (intermediate) cell population (SSC-A/CD172a) does not express SLA Class-II and CD80/86 in both frhBMDC and cryoBMDC cell cultures. (TIF) [file pone.0233773.s003.tif]

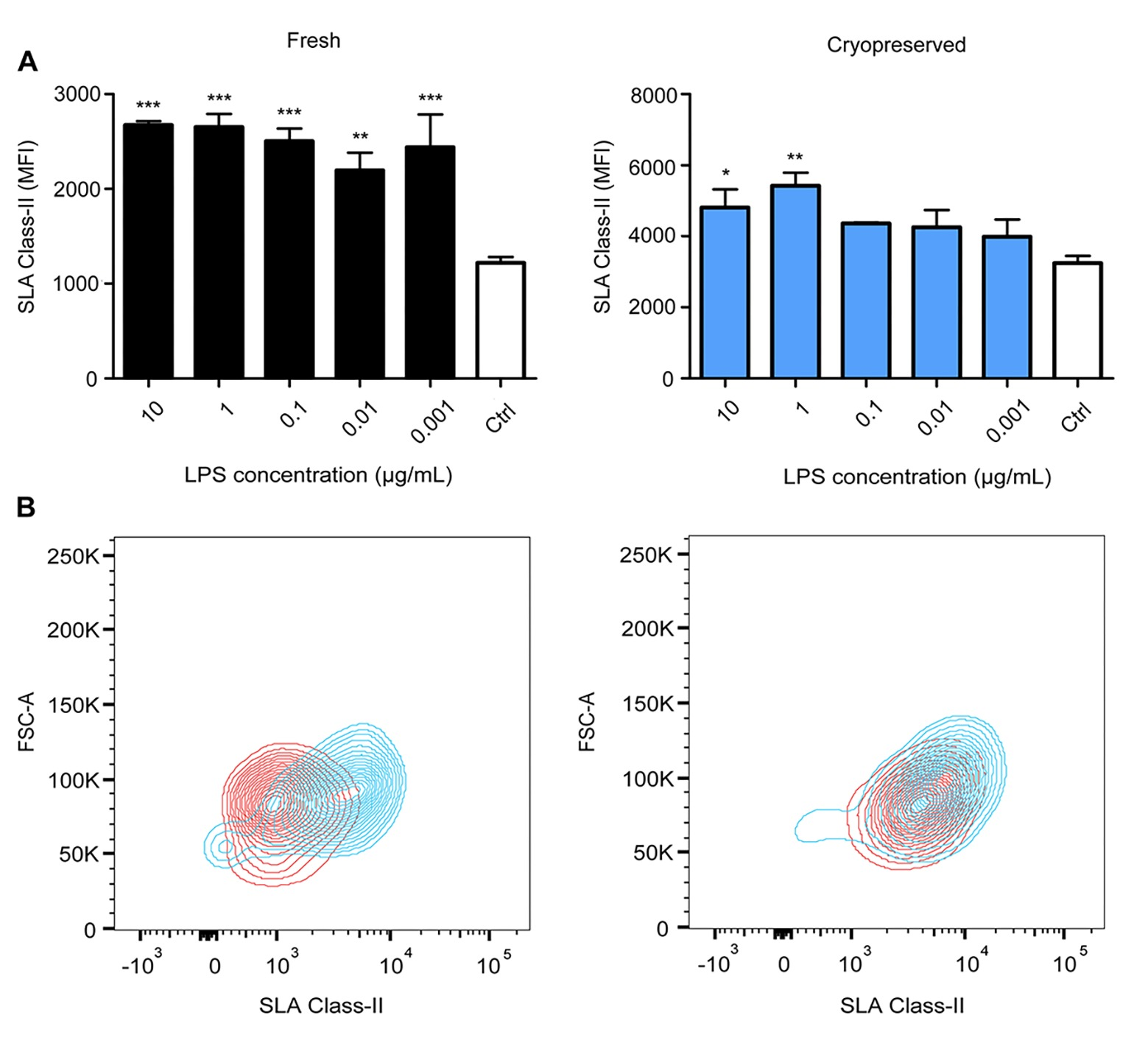

Supplement: S4 Fig — (A) FrhBMDCs and cryoBMDCs (obtained from the same animal, n = 1) were stimulated with different concentrations of LPS or unstimulated using cell culture medium (negative control; Ctrl). After 24 hours, the expression (MFI) of the maturation markers SLA Class-II were measured using Flow Cytometry. The data are shown as the means ± the standard error of the mean (SEM) of three technical replicates. A one-way ANOVA with a Dunnett’s post hoc test was performed, comparing multiple groups to the untreated cells (control): *** = P<0.001, **P<0.01 and * P<0.05. (B) Representative contour plots of SLA Class-II expression on LPS stimulated frhBMDCs and cryoBMDCs. The contour plots are based on forward scatter (y-axis) and SLA Class-II expression (x-axis). The highest concentration of LPS (10 μg/mL) and cell culture medium (negative control; blue) are presented in this figure. (TIF) [file pone.0233773.s004.tif]

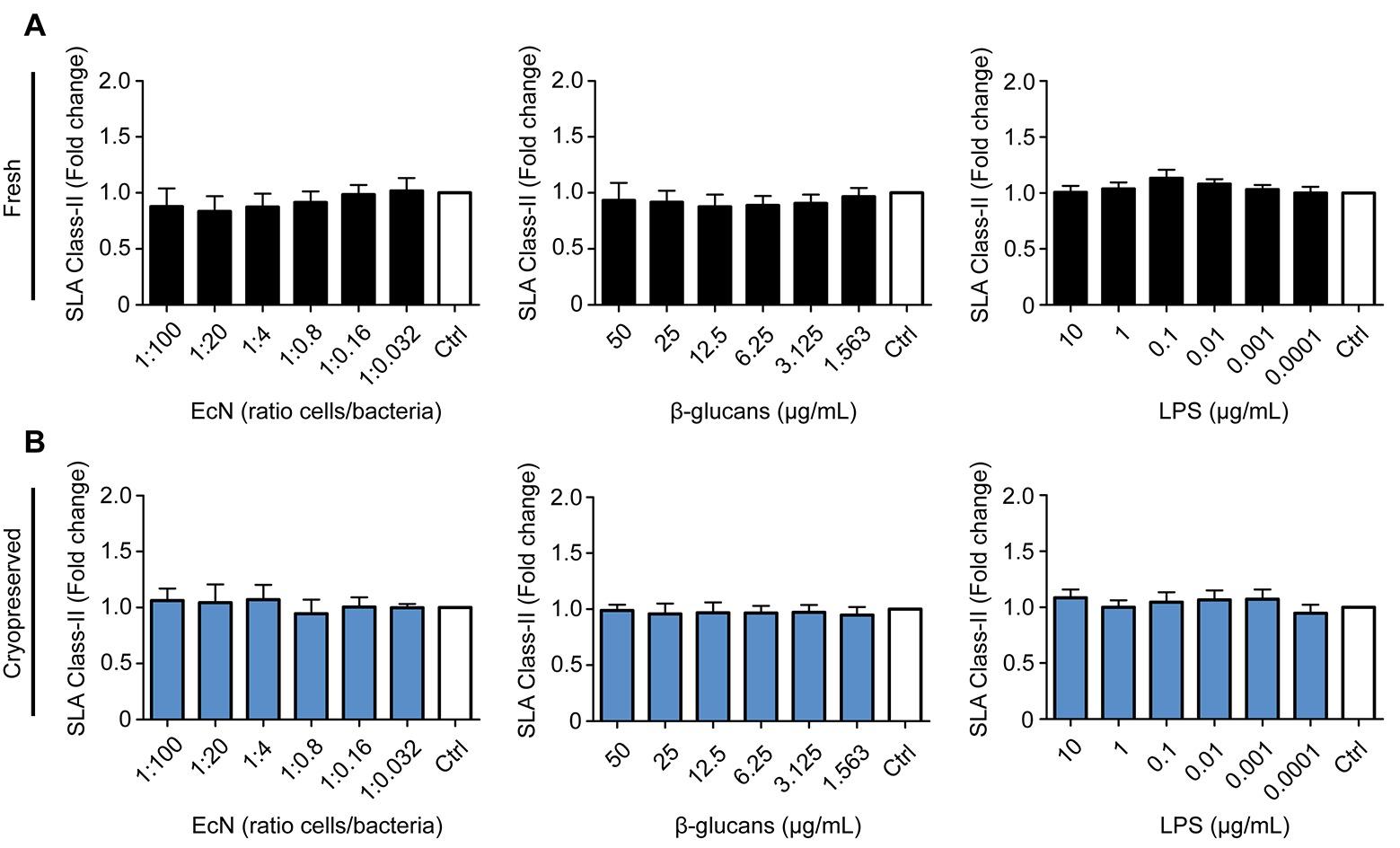

Supplement: S5 Fig — Immature (A) frhBMDCs and (B) cryoBMDCs (obtained from the same animal) were stimulated with different concentrations of E. coli Nissle 1917, β-glucans or LPS. Unstimulated cells are represented by the white bars (negative control; Ctrl). After 24 hours, the upregulation of SLA Class-II was measured using Flow Cytometry (n = 4 animals). Relative fold change was calculated by dividing the MFI of stimulated BMDC/MFI of unstimulated BMDC (Ctrl) of each animal. The data are shown as the means ± the standard error of the mean (SEM) of 4 animals. A one-way ANOVA with a Dunnett’s post hoc test was performed, comparing multiple groups to the untreated cells (control): *** = P<0.001, **P<0.01 and * P<0.05. (TIF) [file pone.0233773.s005.tif]
